# Development of a risk assessment profile tool to determine appropriate use of SARS-CoV-2 rapid antigen detection tests for different activities and events in Ireland, since October 2021

**DOI:** 10.2807/1560-7917.ES.2022.27.3.2101202

**Published:** 2022-01-20

**Authors:** Patrick WG Mallon, Mary Horgan, Conor G McAloon, Peter D Lunn, Julian Little, Andrew Beck, Alexandria Bennett, Nicole Shaver, Aileen McConway, Rhea O’Regan, Barbara Whelan, Rapid Testing Expert Advisory Group, Ireland, Members of the Rapid Testing Expert Advisory Group, Mary Horgan, Jeff Connell, Pete Lunn, Patrick Mallon, Kingston HG Mills, Patrick O’Mahony, Anna-Rose Prior, Breda Smyth

**Affiliations:** 1 Centre for Experimental Pathogen Host Research (CEPHR), University College Dublin, Dublin, Ireland; 2 School of Medicine, University College Cork and Cork University Hospital, Cork, Ireland; 3 School of Veterinary Medicine, University College Dublin, Belfield, Dublin, Ireland; 4 Behavioural Research Unit, Economic and Social Research Institute, Dublin, Ireland; 5 Knowledge Synthesis and Application Unit, School of Epidemiology and Public Health, Faculty of Medicine, University of Ottawa, Ottawa, Canada; 6 Evidence Synthesis Ireland, School of Nursing and Midwifery, National University of Ireland Galway, Galway, Ireland; 7 The members of the RTEAG who were involved in writing this paper are listed under Investigators

**Keywords:** rapid antigen detection test, Ag-RDT, RADT, SARS-CoV-2, Covid-19, risk assessment, Ireland

## Abstract

We describe the development of a risk assessment profile tool that incorporates data from multiple domains to help determine activities and events where rapid antigen detection tests (Ag-RDT) could be used to screen asymptomatic individuals to identify infectious cases as an additional mitigation measure to reduce transmission of SARS-CoV-2. The tool aims to stratify, in real time, the overall risk of SARS-CoV-2 transmission associated with common activities and events, and this can be matched to an appropriate Ag-RDT testing protocol.

Early detection of infection with severe acute respiratory syndrome coronavirus 2 (SARS-CoV-2), in particular of infectious individuals, is key to controlling onward transmission. Timely and accurate testing has been essential to mitigate the coronavirus disease (COVID-19) pandemic. Molecular assays (typically PCR) have been considered the 'gold standard' for detecting cases. However, compared with molecular tests, rapid antigen detection tests (Ag-RDT) offer significantly shorter turnaround time, reduced need for laboratory infrastructure and reduced cost, thus providing an additional mitigation measure in the pandemic response [1]. 

When to use Ag-RDT to reduce onward transmission of SARS-CoV-2 requires careful consideration. Factors to consider include the demographics of the target population, the risk for attendees in a setting being infected with SARS-CoV-2 and the risks and consequences of transmission within a given environment. We present the development of a risk assessment profile tool to guide appropriate use of Ag-RDT for screening asymptomatic individuals engaging in common events and activities, to inform the public health approach to the COVID-19 pandemic in Ireland. The risk assessment profile tool aims to stratify, in real time, the overall risk of SARS-CoV-2 transmission associated with different settings. The tool is recommended in addition to other public health measures.

## Risk assessment profile tool to evaluate an appropriate Ag-RDT testing practice

The risk assessment profile tool is in an updateable format that can be regularly modified based on changes in the epidemiology of infection and emerging evidence around the risk of SARS-CoV-2 transmission and Ag-RDT use.

The first task was to generate a ‘list of common events and activities’ deemed strategically important to government departments in Ireland, and which were important from a preparedness perspective when reopening society as restrictions were relaxed. Events were defined as organised infrequent gatherings of limited duration that bring people together, while activities were defined as regular gatherings of people for a defined purpose. Subsequently, we derived an estimate of the population size and age distribution of attendees for each of the common activities using data from a fortnightly national behavioural survey, the Social Activity Measure (SAM), designed by the Economic and Social Research Institute’s Behavioural Research Unit, in consultation with Ireland’s Department of the Taoiseach [2]. Where SAM data were unavailable, we derived estimates through consensus discussion with government departments with detailed knowledge of the different sectoral activities.

The generation of a recommendation for each activity or event proceeded in four steps.

### Step 1. Provide a graded risk of the consequences of transmission at a specific activity or event

The risk of severe disease and mortality from COVID-19 increases with co-morbidities, being immunocompromised, not being vaccinated and with older age [3]. The risk in this step is assessed based on the median age of attendees, derived from the population estimates described above. This step assigns one of three risk categories; low (expected median age: < 45 years), medium (expected median age: 45–65 years) and high (expected median age: > 65 years). While Step 1 would ideally consider all factors associated with consequences of infection, the goal was to develop a tool for practical use across a population. Furthermore, as data on prevalence of comorbidities and immunocompetence are not readily available for the general population in relation to attendance at specific events, we chose age of attendees as the most relevant factor that could be incorporated into a tool that would provide clinically relevant and applicable data on risk of onward transmission. Similarly, for simplicity, and to take a conservative approach to assigning risk, vaccination rates by age were not included in this step, but are inherently included in Step 2, as the time-updated background prevalence of SARS-CoV-2 estimated from regularly updated local data would reflect an impact of vaccination within the population from which the data were derived. 

### Step 2. Provide a graded risk for attendees being infected with SARS-CoV-2 and capable of onward transmission to other attendees

The risk is estimated based on prevalence likelihoods derived from real-time data on the epidemiology of COVID-19 in Ireland, including age-related case notifications, enabling regular (e.g. weekly) updates as the background epidemiological picture in Ireland changes. Estimated prevalence is assigned one of three risk categories (Table 1), which are broadly in line with the categorisation of high prevalence (> 10% prevalence) proposed by the European Centre for Disease Prevention and Control [4].

**Table 1:** Categories of risk of SARS-CoV-2 infection for risk assessment Step 2, Ireland, October 2021

Step 2 risk category	Estimated prevalence of infectious SARS-CoV-2 within participants/attendees
Low	≤ 10:1,000 (≤ 1%)
Medium	> 10:1,000 to < 100:1,000 (> 1% to < 10%)
High/very high	≥ 100:1,000 (≥ 10%)

SARS-CoV-2: severe acute respiratory syndrome coronavirus 2.

### Step 3. Provide an overall estimate of the risk of transmission associated with an activity or event

Updated estimates of this risk come from a living rapid review on the risk of SARS-CoV-2 transmission for different activities and events (e.g. restaurants, bars, live entertainment, conferences, sports events, exercise classes), currently being conducted by Beck et al. [5]. Updated monthly, the review findings are used to assign a risk category of low, moderate or high for the various activities and events. Where evidence is not yet available, risk is assigned based on consensus expert opinion on the risk associated with similar settings for which evidence exists. The review is ongoing, expedited draft summaries are available [6] and the final report will be published once completed.

### Step 4. Attribute an antigen testing practice to each activity or event

Each activity or event is assigned one of four Ag-RDT testing protocols (no additional testing recommended, single self-test, single supervised test, twice weekly self-test or twice weekly supervised test). Only Ag-RDT with a CE mark on the packaging are recommended for use. The Ag-RDT testing protocols are graded in order of their likelihood to capture an infectious case and broken down into whether someone is attending an event or activity. The evidence supporting these grades comes from a living rapid review on COVID-19 rapid antigen testing strategies [7], which is updated monthly and explores the effectiveness of different testing strategies (self-administered vs supervised testing and/or single test vs serial testing) for reducing transmission or detecting infectious cases. Findings to date [6] inform the testing protocols recommended in Tables 2 and 3. Of the 500 full-text articles reviewed so far, four assessed self-sampling (supervised and non-supervised) vs professionally collected nasal samples [8-11] and, overall, yielded comparable results. The review is ongoing and will be published once completed. For each specific activity or event, data from the four steps populate a grid (Table 2 for events and Table 3 for activities).

**Table 2:** SARS-CoV-2 risk assessment profile tool for attendance at an event, Ireland, November 2021

Step 1	Step 2	Step 3	Step 4
Population risk of severe disease	Estimated prevalence of COVID-19 at event	Risk of transmission associated with event	Minimum testing recommended in addition to standard public health measures
Low (expected median age: < 45 years)	Low	Any^a^	No additional testing
	Medium	Low	
		Moderate	Single self-test
		High	
	High/very high	Low	
		Moderate	Single self-test
		High	Single self-test
Medium (expected median age: 45–65 years)	Low	Low	No additional testing
		Moderate	
		High	Single self-test
	Medium	Low	No additional testing
		Moderate	Single self-test
		High	Single self-test
	High/very high	Low	Single self-test
		Moderate	Single self-test
		High	Single supervised test
High (expected median age: > 65 years)	Low	Low	No additional testing
		Moderate	Single self-test
		High	
	Medium	Low	
		Moderate	Single self-test
		High	Single self-test
	High/very high	Low	Single self-test
		Moderate	Single supervised test
		High	Single supervised test

COVID-19: coronavirus disease; SARS-CoV-2: severe acute respiratory syndrome coronavirus 2.

^a^ Represents any risk category of transmission related to an event (low–high).

**Table 3:** SARS-CoV-2 risk assessment profile tool for participation in an activity, Ireland, November 2021

Step 1	Step 2	Step 3	Step 4
Population risk of severe disease	Estimated prevalence of COVID-19 at activity	Risk of transmission associated with activity	Minimum testing recommended in addition to standard public health measures
Low (expected median age: < 45 years)	Low	Any^a^	No additional testing
	Medium	Low	
		Moderate	Single self-test
		High	
	High/very high	Low	
		Moderate	Twice weekly self-test or single supervised test
		High	Twice weekly self-test or single supervised test
Medium (expected median age: 45–65 years)	Low	Low	No additional testing
		Moderate	
		High	Single self-test
	Medium	Low	No additional testing
		Moderate	Single self-test
		High	Twice weekly self-test or single supervised test
	High/very high	Low	Single self-test
		Moderate	Twice weekly self-test or single supervised test
		High	Twice weekly supervised testing
High (expected median age: > 65 years)	Low	Low	No additional testing
		Moderate	Single self-test
		High	
	Medium	Low	
		Moderate	Twice weekly self-test or single supervised test
		High	Twice weekly self-test or single supervised test
	High/very high	Low	Twice weekly self-test or single supervised test
		Moderate	Twice weekly supervised testing
		High	Twice weekly supervised testing

COVID-19: coronavirus disease; SARS-CoV-2: severe acute respiratory syndrome coronavirus 2.

^a^ Represents any risk category of transmission related to an event (low–high).

## Final output from risk assessment profile tool

A risk assessment profile is presented (Table 4) for example events (attending the theatre and a wedding) and activities (going to a restaurant and bar) based on data available on 24 November 2021. These recommendations can be updated regularly with changes to the background epidemiological patterns of COVID-19 within the community and the availability of new research to guide recommendations. They can then be communicated to government departments and the public.

**Table 4:** Example of a SARS-CoV-2 risk assessment for events and activities, Ireland, based on the situation on 24 November 2021

Event/activity (number of attendees)	Step 1 Population risk of severe disease	Step 2 Estimated prevalence of COVID-19 at event (per 1,000)	Step 3 Risk of transmission from event/activity (based on living rapid review^a^)	Step 4 Minimum testing recommended in addition to standard public health measures (based on living rapid review^b^)
Going to the theatre (n = 500)	Low (expected median age: < 45)	13.65 (medium)	Moderate	Single self-test
Going to a wedding (n = 100)	Low (expected median age: < 45)	13.21 (medium)	High	Single self-test
Going to a restaurant (n = 150)	Low (expected median age: < 45)	13.26 (medium)	High	Single self-test
Going to a bar (n = 150)	Medium (expected median age: 45–65 years)	13.22 (medium)	High	Twice weekly self-test or single supervised test

COVID-19: coronavirus disease; SARS-CoV-2: severe acute respiratory syndrome coronavirus 2.

a See Living Rapid Review Risk Activities Preliminary Report #2_2021-11-15 [6].

^b^ See Living Rapid Review Antigen Testing Preliminary Report #1_2021-11-08 [6].

## Discussion

Testing strategies for SARS-CoV-2 vary widely between countries [12]. One strategy for using Ag-RDT is to have proactive localised screening to detect the highest number of asymptomatic infectious cases at the lowest cost [13]. This risk assessment profile tool facilitates a focused approach based on assessments of risk derived from current national epidemiology and international published research. This tool has been used by the Rapid Testing Expert Advisory Group to advise the Department of Health in Ireland on the best Ag-RDT testing strategy for different events and activities and to identify settings associated with a higher-risk profile. Following this, the Government of Ireland has recommended that people who are asymptomatic and engage in activities with a high-risk profile (as identified by the tool) undertake twice weekly antigen tests [14].

This approach does have limitations. While Ag-RDT have lower sensitivity than molecular tests, their specificity is generally high, and they are sensitive enough to detect individuals who have high viral loads. Given reduced sensitivity, Ag-RDT need to be used in combination with other public health measures as a layered approach to reduce risk of transmission [15]. Furthermore, because of lower positive predictive values in settings of low pre-test probability [4], a confirmatory molecular test or higher specificity Ag-RDT testing is recommended [16]. One systematic review [17] (Test 5, page 318) showed very high sensitivity of Ag-RDT in samples with quantification cycle (Cq) values associated with potentially infectious individuals (≤ 25 overall). 

The risk assessment tool presented here aims to prevent onward transmission during an activity or event that is imminent by deploying Ag-RDT to identify and remove infectious individuals from that event, even if asymptomatic. Although data are derived from updated evidence syntheses and epidemiological data, they do not incorporate updated risk assessments that may accompany a new virus variant. For example, with the introduction of the Omicron variant, although this would not impact on real-time evaluation of estimated prevalence, given its higher attack rate, risk of transmission associated with activities or events would probably be increased. Some settings in Step 3 (such as going to the theatre) would therefore carry a higher risk of transmission. Such limitations will be inherent in any risk assessment that is evidence-based, given the lag between the introduction of new variants and the relevant evidence being produced to guide transmission risk. These limitations are partly overcome by our approach of using an ongoing ‘living’ evidence synthesis, which ensures up to date data are incorporated into the overall risk assessment as they become available. 

The proposed risk assessment tool also does not incorporate unmeasured behavioural factors that may alter transmission risk or unmeasured prevalence of comorbidities or immunosuppression within specific populations that may impact on the consequences of onward transmission associated with an event or activity. These factors are pertinent given the emergence of the SARS-CoV-2 Omicron variant of concern. Some risk categories in the tool may need to be adapted as evidence accumulates. In addition, where community transmission rates are so high that regular antigen testing is recommended for everyone as part of standard public health measures, the tool would not be relevant. However, as waves of infection rise and recede, the tool can serve as an indicator for both escalation and de-escalation of the use of Ag-RDT as community transmission rates change. For the estimate of risk in Step 2, vaccination rates are not explicitly included but the case count is used which is affected by vaccination rates. 

This risk assessment profile tool has been developed in one country and should ideally be validated prospectively and in multiple geographical areas. However, the model has been updated on a weekly basis since October 2021 and the testing strategy recommended for settings of interest has changed as the incidence of COVID-19 has increased in Ireland. Hence the model can discern the level of risk based on real-time epidemiology. Although further research is needed to assess the tool’s capacity to prevent transmission of SARS-CoV-2, the outputs from the tool have fed into recent recommendations in Ireland that individuals who are participating in high-risk activities undertake antigen testing twice weekly to reduce the transmission of SARS-CoV-2.

## Conclusion

This risk assessment profile tool helps to target activities and events where Ag-RDT could be used to screen asymptomatic people to reduce onward transmission of SARS-CoV-2. By using real-time data, the tool adapts to the dynamic nature of the COVID-19 pandemic and provides a focused, scientific approach to using Ag-RDT for people engaging in different activities and events.
