# Author's correction for Euro Surveill. 2022;27(3)

**DOI:** 10.2807/1560-7917.ES.2021.27.6.210310c

**Published:** 2022-02-10

**Authors:** Eurosurveillance editorial team

**Affiliations:** 1 European Centre for Disease Prevention and Control (ECDC), Stockholm, Sweden

In the article ‘Development of a risk assessment profile tool to determine appropriate use of SARS-CoV-2 rapid antigen detection tests for different activities and events in Ireland, since October 2021’ by Mallon et al., published on 20 January 2022, the name of author Aileen Conway was corrected (erroneously written as Aileen McConway in the original publication). This change was made on request of the authors on 4 February 2022.

